# Metagenomic Analysis of Antibiotic Resistance Genes in Dairy Cow Feces following Therapeutic Administration of Third Generation Cephalosporin

**DOI:** 10.1371/journal.pone.0133764

**Published:** 2015-08-10

**Authors:** Lindsey Chambers, Ying Yang, Heather Littier, Partha Ray, Tong Zhang, Amy Pruden, Michael Strickland, Katharine Knowlton

**Affiliations:** 1 Department of Dairy Science, Virginia Polytechnic Institute and State University, Blacksburg, Virginia, United States of America; 2 University of Hong Kong, Hong Kong, China; 3 Department of Civil and Environmental Engineering, Virginia Polytechnic Institute and State University, Blacksburg, Virginia, United States of America; 4 Department of Biological Sciences, Virginia Polytechnic Institute and State University, Blacksburg, Virginia, United States of America; U. S. Salinity Lab, UNITED STATES

## Abstract

Although dairy manure is widely applied to land, it is relatively understudied compared to other livestock as a potential source of antibiotic resistance genes (ARGs) to the environment and ultimately to human pathogens. Ceftiofur, the most widely used antibiotic used in U.S. dairy cows, is a 3^rd^ generation cephalosporin, a critically important class of antibiotics to human health. The objective of this study was to evaluate the effect of typical ceftiofur antibiotic treatment on the prevalence of ARGs in the fecal microbiome of dairy cows using a metagenomics approach. β-lactam ARGs were found to be elevated in feces from Holstein cows administered ceftiofur (n = 3) relative to control cows (n = 3). However, total numbers of ARGs across all classes were not measurably affected by ceftiofur treatment, likely because of dominance of unaffected tetracycline ARGs in the metagenomics libraries. Functional analysis via MG-RAST further revealed that ceftiofur treatment resulted in increases in gene sequences associated with “phages, prophages, transposable elements, and plasmids”, suggesting that this treatment also enriched the ability to horizontally transfer ARGs. Additional functional shifts were noted with ceftiofur treatment (e.g., increase in genes associated with stress, chemotaxis, and resistance to toxic compounds; decrease in genes associated with metabolism of aromatic compounds and cell division and cell cycle), along with measureable taxonomic shifts (increase in Bacterioidia and decrease in Actinobacteria). This study demonstrates that ceftiofur has a broad, measureable and immediate effect on the cow fecal metagenome. Given the importance of 3^rd^ generation cephalospirins to human medicine, their continued use in dairy cattle should be carefully considered and waste treatment strategies to slow ARG dissemination from dairy cattle manure should be explored.

## Introduction

In addressing the global problem of antibiotic resistance, the livestock industry and its use of antibiotics is attracting growing attention. In 2013, the FDA reported that 14.8 million kg of antibiotics were sold for use in domestic livestock, with 28 thousand kg being cephalosporins [1]. The prevalent antibiotic use in livestock, combined with a high excretion rate of administered antibiotics [2] and a reservoir of resistant gut bacteria, may have synergistic impacts on affected soil and water environments. Prior to fecal excretion, ARGs have the potential to be horizontally transferred among bacterial species within the animal gut and subsequently with environmental bacteria when fecal material is land-applied [3]. Runoff from farms has been shown to contain higher levels of ARGs than other nearby water sources [4] and land application of contaminated cattle feces to vegetable plots has been documented to be capable of carrying over ARGs onto produce [5]. Several studies have demonstrated higher levels of antibiotic resistance genes (ARGs) in surface waters influenced by livestock facilities [4,6,7]. Through environmental sources such as these, ARGs excreted in livestock manure can potentially reach the human populations

Use of drugs in livestock corresponding to important human antibiotics is especially of concern. In particular, The World Health Organization has deemed 3^rd^-generation cephalosporins to be critically important antibiotics, meaning that they (1) serve as the sole or limited alternative for treating serious human infections and (2) are used to treat bacterial diseases capable of transmission from non-human sources [8]. The cephalosporin drug class is the second largest antibiotic class used in the human pharmaceutical industry, comprising 15.1% of the total purchased antibiotics in 2012 [9]. In cattle, a 3^rd^-generation cephalosporin, ceftiofur, is the most widely used antibiotic for the treatment of common ailments such as respiratory disease, foot rot, and metritis (uterine infections) [10] because it is not secreted into the milk and so requires no withholding time before the milk is sold. Thus, there is legitimate concern that the routine use of ceftiofur in cattle could enrich β-lactamase genes and other ARGs that impart resistance to a variety of antibiotics critically important to human health. However, the potential for ceftiofur to select for broad suites of ARGs in the cattle manure microbiome has not previously been examined.

Dairy manure is of particular interest, as it is widely applied as a soil amendment, but has received relatively less attention than other livestock manures. Prior studies have examined the effects of conventional antibiotic use practices (e.g., use of ceftiofur and cephalosporin) on dairy farms, relative to organic practice (i.e., no antibiotic use) on antibiotic resistance patterns of specific bacteria of interest, such as *Campylobacter* spp. and *Escherichia coli* [11–13]. Generally, higher proportions of isolates from conventional dairy farms tend to be resistant to many, but not all, of the antibiotics examined. Interestingly, while increases in resistance of cow fecal bacteria during antibiotic treatment have been observed, often the increase is transient (2–13 d) [14–17]. It is also important to note that even cattle never exposed to antibiotics shed antibiotic resistant bacteria [18,19]. However, prior studies have not provided a comprehensive understanding of the impacts of antibiotic treatment on a broad range of ARGs and the corresponding bovine intestinal flora. In general, there is a need for controlled studies to comprehensively examine the effect of administered antibiotics on ARGs and associated horizontal gene transfer elements in the context of the corresponding taxonomic composition [20].

Most recently, metagenomic approaches, such as direct shot-gun 454 pyrosequencing [21] and Illumina sequencing [22] of extracted DNA, are a means by which previous methodological disadvantages can be overcome. Metagenomics allows direct access to the total DNA present in a sample, with the resulting output representative of multiple bacterial genomes, without the need for *a priori* selection of target genes of interest. Metagenomic data can be annotated using bioinformatics databases such as MG-RAST (Meta Genomics Rapid Annotation using Subsystem Technology)[23] and other specialized curated databases, allowing broad capture of elements associated with antibiotic resistance (e.g. antibiotic resistance database (ARDB) [24] and the comprehensive antibiotic resistance database (CARD) [25]. Together these methods provide a depth and breadth of analysis not previously realized using targeted approaches, such as culturing or quantitative polymerase chain reaction (qPCR).

The purpose of this study was to examine the effect of typical ceftiofur treatment on the occurrence of ARGs in cow feces in the broader context of the fecal microbiome using a metagenomics approach. Such information is needed to inform appropriate management options, such as limiting use of 3^rd^ generation cephalosporins or appropriate segregation/treatment of corresponding manure.

## Materials and Methods

### Ethics Statement

This experiment was conducted under the review and approval of the Virginia Tech Institutional Animal Care and Use Committee (protocol 12-184-DASC).

### Animals and Experimental Treatments

Holstein (n = 6) cows 110–200 days into their first lactation and yielding 30.5 to 40.5 kg of milk daily, were selected from the Virginia Tech Dairy Center (Blacksburg, VA). These cows had not received any antibiotic treatment for at least nine months prior to parturition (the time of insemination). Cows were housed individually in tie-stalls (1.25 × 2.25 m) with rubber mats, fed a total mixed ration twice daily, and provided *ad libitum* access to water.

Six cows were randomly assigned to the control (no antibiotics, n = 3) or antibiotic treatment (n = 3) groups using a random number generator. Cows assigned to the antibiotic treatment were injected subcutaneously with 1.5 mL (150 mg ceftiofur) ceftiofur crystalline free acid sterile suspension (Excede, Zoetis, Madison, NJ) per 45.4 kg body weight. Per manufacturer’s protocol, a sequence of two injections were spaced 72 hours apart, at the base of the right ear on day 0 and at the base of the left ear on day 3. Prior to injection, the area was cleaned with a 4 × 4 gauze pad soaked in 70% isopropyl alcohol.

### Sample Collection

Fecal samples were collected from all cows 30 min prior to antibiotic administration on day 0 (at 1730 h) and then on day 3 following treatment (at 1800 h). Fecal samples were collected rectally, using a clean palpation sleeve and sterile lubricant for each collection. To prevent contamination, approximately 500 g of feces was discarded before 100 g of wet sample was collected in a sterile plastic vial. Fecal samples were stored at -20°C within one hour of collection for future analysis.

### Sample Preparation and DNA Extraction

Fecal samples were thawed and a subsample of 15 g was freeze-dried (LABCONCO, Kansas City, MO) for 72 hours or until the expected dry matter content (13–19% dry matter) was achieved. For DNA extraction, 0.102 g of freeze-dried fecal samples were extracted in replicates of 6 using a QIAamp DNA Mini Stool Kit (QIAGEN, Germantown, MD) following the manufacturer’s Stool Pathogen Detection Protocol with some modifications. First, the ASL Buffer (lysis buffer) was heated (45–50°C) prior to use until precipitates dissolved. In the lysis step (step 3), samples were heated at 95°C for 6 minutes to improve lysis of Gram-negative bacteria. In step 8, prior to centrifugation, 4 μL of RNase A (Epicentre, Madison, WI) (conc. of 5 μg/μL) was added, the tube was inverted to mix, and the supernatant was allowed to incubate for 3 min at room temperature. The purpose of the RNase A was to rid the sample of any RNA contamination prior to metagenomics sequencing. The recommended step 17 of centrifugation for 1 min with a new catch tube was included. In step 18 (elution step), 50 μL of Buffer EB (10 mM Tris-Cl) was used because it does not contain EDTA, which inhibits Illumina library preparation. Also, the Buffer EB was heated at 60°C for 2 min prior to its addition to the column and was allowed to incubate on the column for 6 min prior to centrifugation; this aided in the binding of the buffer to the DNA on the column. Finally, all centrifugation steps were carried out at 16,100 × *g*. The eluted DNA, consisting of both intracellular and extracellular since DNase was not applied, was immediately stored at -20°C until further processing.

### Sample Purification and Quality Testing

The DNA concentration of each sample replicate (n = 6) was quantified using a Qubit 2.0 Fluorometer (Life Technologies, Grand Island, NY) with the Qubit dsDNA HS Assay Kit. Samples were submitted to the Virginia Bioinformatics Institute Genomics Research Laboratory (Blacksburg, VA) for Solid Phase Reversible Immobilization (SPRI) Purification, using Agencourt AMPure XP. This purification process uses magnetic beads to bind DNA fragments larger than 100 bp, ridding the sample of contaminants like salts or primer dimers (Beckman Coulter, Inc., 2013). After purification, the DNA concentrations of each sample replicate was again quantified using a Qubit 2.0 Fluorometer with the Qubit dsDNA HS Assay Kit. Quality of sample replicates was assessed by agarose gel electrophoresis. Lastly, sample replicates were analyzed on a NanoPhotometer (Implen, Inc., Westlake Villiage, CA) to confirm that the 260/280 value of each replicate was approximately 1.8, indicative of DNA purity. The two replicates of each sample best meeting quality criteria, determined by 260/280 ratio, highest DNA concentration, and gel images indicative of intact DNA, were pooled and submitted for library preparation and sequencing.

### Library Preparation and Illumina GAIIx HiSeq Sequencing

DNA Seq library preparation and Illumina paired-end HiSeq, 100 cycle, 101 bp read length, multiplexed sequencing was performed by the Virginia Bioinformatics Institute Genomics Research Laboratory (Blacksburg, VA). HiSeq sequencing was performed on day 0 (pre-treatment) and day 3 (post-treatment) samples for all control and ceftiofur cows (a total of 12 samples sequenced). HiSeq sequencing resulted in an average of 29 million raw reads per sample that passed the Illumina chastity filter, a filtering method that reduces the amount of unreliable data due to potential miscalls.

### BLAST/ARDB Data Analysis

BLASTX was conducted using E-value cutoff of 1e-5 to identify ARG-like sequences among the Illumina sequences against the ARDB [24,26]. A matched sequence from BLASTX was further annotated as ‘ARG-like’ if its best hit in the ARDB reached a threshold of 90% amino acid sequence identity and alignment length of at least 25 amino acids [27,28]. The classification of ARG-like sequences was performed using the structured database of ARDB and customized script as described previously [26].

### MG-RAST Analysis

Sequence analysis on the 12 paired-end samples was performed using MG-RAST. The paired-end sample sequences were obtained from the Virginia Bioinformatics Institute (VBI, Blacksburg, VA) as two individual files, one file per end. The two single ends of the paired-end sample sequences were uploaded, in fastq format, to MG-RAST and any bovine host sequences (those associated with the *Bos taurus* genome) were removed. The remaining sequences were quality checked and de-replication was performed to remove false sequences. Of the sequences that passed these quality tests (roughly 90% of the original 29 million sequences), predicted protein coding regions were determined, assigned annotation based on SEED sequence identification, and assigned to a functional category using the SEED subsystems. On average, 6–9 million sequences for each sample were assigned a SEED function. Functional data for each of the single-end sequences was downloaded from MG-RAST and merged to create paired-end results for each sample prior to statistical analysis.

### Statistical Analysis

Statistical analysis of BLAST/ARDB and MG-RAST data was carried out using the PROC GLIMMIX test as implemented by SAS 9.2 (SAS Institute Inc. Cary, NC). A minimum of 40 or more sequences per sample was set as a threshold for ARGs to be analyzed in the BLAST/ARDB dataset. Samples from day 0 were used as a covariate.

Permutational MANOVA (perMANOVA) was used to examine differences between whole bacterial community composition, metagenome composition, and the composition of genes corresponding to resistance to antibiotics and toxic compounds (RATC) SEED subsystem. Treatments, blocked by cow, were designated as control or ceftiofur, collected on day 0 or day 3, and analyses were conducted on Bray-Curtis distance matrixes. Within treatments, homogeneity of multivariate dispersions was also tested, which allowed for identification of the role of specific treatments in community variability or metagenome composition. Additionally, the dissimilarity in community or metagenome composition was determined by calculating the change in Bray-Curtis distance after 3 days of exposure to the control or ceftiofur treatment. These calculations enabled examination of treatment effect on change in community or metagenome composition, regardless of the response of an individual cow. Results were analyzed via ANOVA and Principal Coordinates Analysis (PCoA) was implemented to visualize the influence of treatment. All perMANOVA analyses, tests of homogeneity, and PCoA were conducted using Primer [29].

Because of known high inter-animal variation, significance was declared at *P* ≤ 0.10 and trends were declared at *P* ≤ 0.15.

## Results and Discussion

### Effect of Ceftiofur on Occurrence of ARGs in Cow Feces

**Fig 1:**
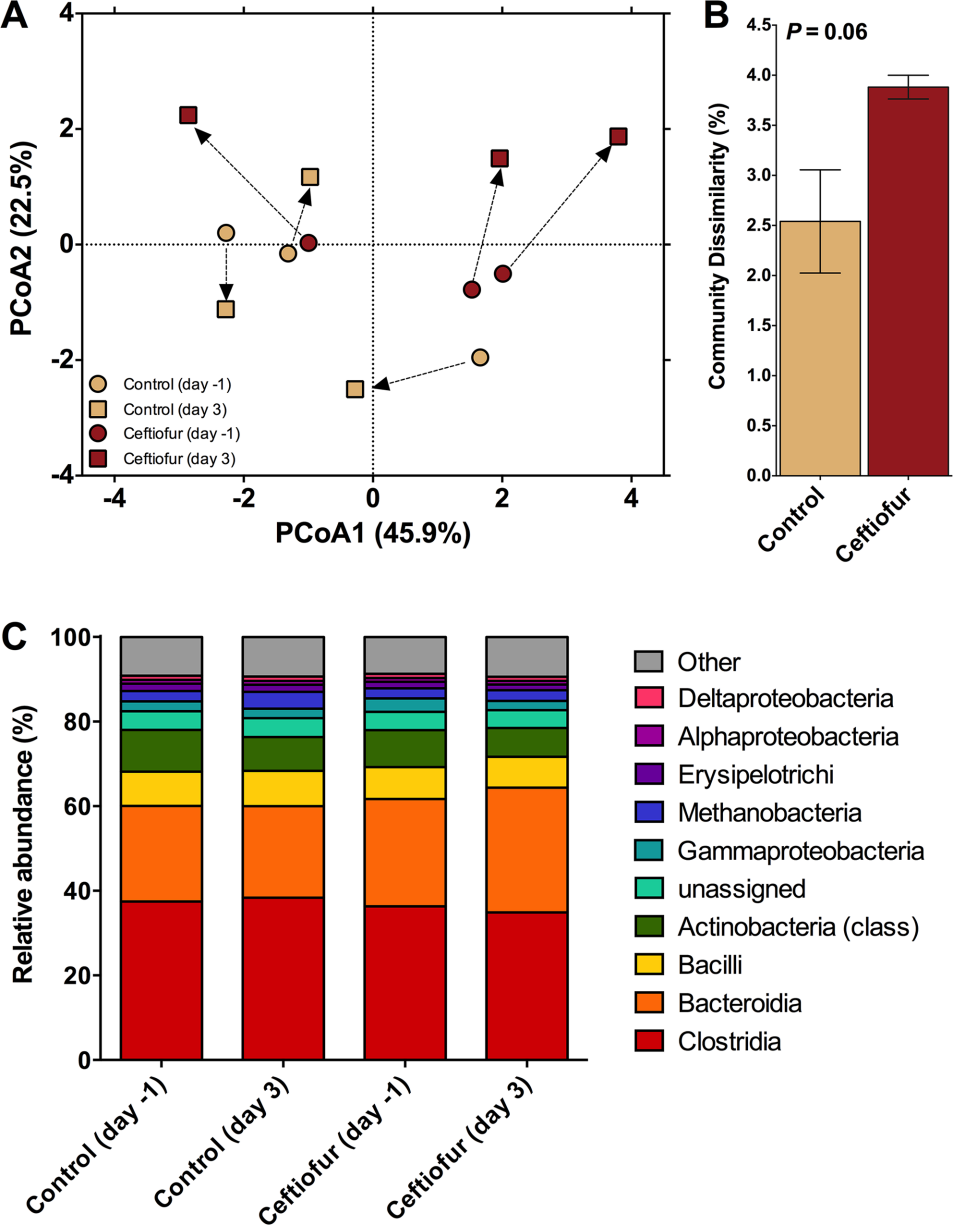
A) Abundance of antibiotic resistance gene (ARG)-like sequences in fecal samples collected from control (n = 3) and ceftiofur crystalline free acid treated (n = 3) cows on 3 post-treatment. Day 0 (pre-treatment) samples were used as a covariate. Values are expressed as a proportion of the total sample sequences (x 10^−6^). There was no effect of antibiotic treatment on the abundance of ARG-like sequences as a proportion of total sample sequences. B) Breakdown of ARG classes as a % of total ARG-like sequences for ceftiofur-treated cows (n = 3) on day 3 post-treatment. The tetracycline ARG class comprised the majority of detected ARGs at 75% of the total ARG-like sequences.

To determine the effect of ceftiofur treatment on fecal occurrence of ARGs, metagenomic data were compared against the ARDB using the approach developed by Yang et al. (2013) to account for redundancies and inaccurate sequences in the database. Overall, there was no measureable effect of ceftiofur treatment on total ARG-like sequence abundance (Fig 1A). This apparent lack of effect is likely explained by the dominance (>85% of the sequences associated with antibiotic resistance) of sequences coding for resistance to tetracyclines in both ceftiofur-treated and control cows. The presence of ARGs in the fecal bacteria of cows with no previous exposure to antibiotics is well-documented, with the largest resistance category being against antibiotics, such as tetracyclines, that attack drug efflux pumps as a major mode of action [18]. For instance, genes coding for tetracycline resistance (*tet*(W) and *tet*(Q)) were detected in more than 80% of fecal samples collected from cattle raised in grassland-production systems without antibiotics [4]. Therefore, given the lack of impact of ceftiofur on tetracycline resistance sequences, their overwhelming presence in the current fecal samples (Fig 1B) may have masked any effect of ceftiofur treatment on abundance of other ARGs. Interestingly, a recent metagenomic study of manure from four dairy cows indicated that the chloramphenicol resistance class was dominant, with the tetracycline resistance the fourth most abundant class [3]. Thus, the composition of the antibiotic resistome may vary from herd to herd.

**Fig 2:**
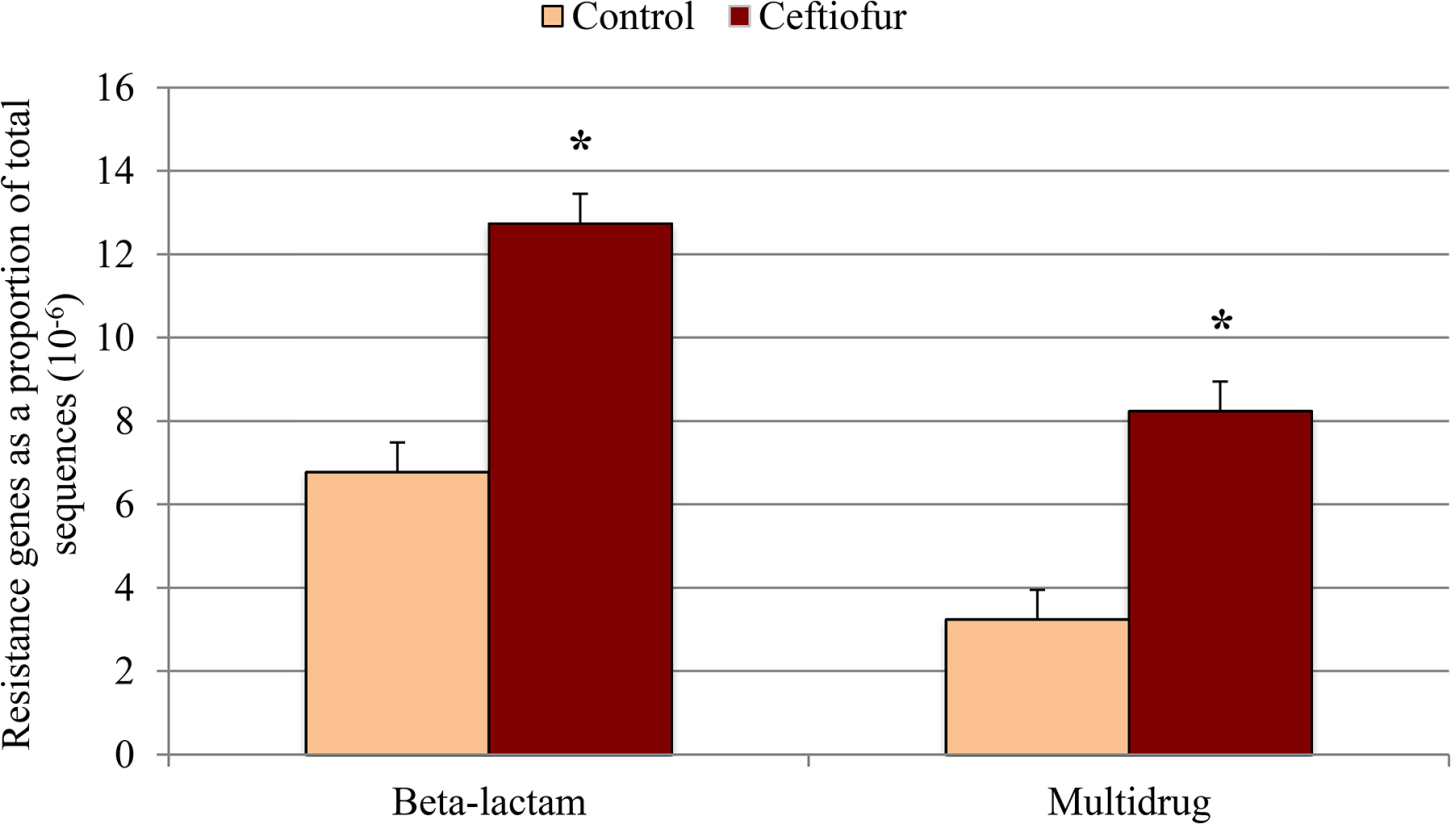
Abundance of β-lactam and multidrug resistance genes in fecal samples collected from control (n = 3) and ceftiofur crystalline free acid treated (n = 3) cows on 3 post-treatment. Day 0 (pre-treatment) samples were used as a covariate. Values are expressed as a percentage of the total sample sequences (x 10^−6^). The symbol * indicates significant (*P* ≤ 0.10).

The metagenomics approach enabled direct and comprehensive evaluation of genes encoding resistance to the β-lactam class of antibiotics, which were of special interest given that ceftiofur belongs to this class. The proportion of β-lactam (*P* < 0.10; Fig 2) and multidrug (*P* < 0.10; Fig 2) ARG sequences were higher in ceftiofur-treated cows relative to control cows. In particular, genes CfxA2 and CfxA3were noted to be the most abundant in the β-lactam class in ceftiofur-treated cow feces, relative to the control. The Class A β-lactamases CfxA2 and CfxA3 have been found in *Prevotella* spp. [30] and *Capnocytophaga* spp. [31], both common culprits of dental diseases in the human health sector. Increase of β-lactam ARGs could be a concern, given the importance of this class of antibiotics to human health.

Higher β-lactam ARGs in ceftiofur-treated cow manure is consistent with a prior study of resistant *E*. *coli* in feedlot steers. When steers were given one dose of ceftiofur (same form as used in current study) at either 4.4 mg/kg body weight or 6.6 mg/kg body weight, fecal shedding of ceftiofur-resistant *E*. *coli* isolates was greater relative to control steers on 2, 6, 9, and 16 days following treatment [17]. In a similar study that used q-PCR to quantify the *bla*_*CMY-2*_ ARG encoding β-lactam resistance, beef steers treated with one dose of 4.4 mg/kg ceftiofur carried higher fecal gene copies of *bla*_*CMY-2*_ on days 3, 7, and 10 post-treatment than controls [32]. In another study, dairy cows treated with five doses of 2.2 mg/kg ceftiofur carried more fecal *E*. *coli* isolates resistant to antibiotics on days 4, 5, and 6 post-treatment than control cows [14]. This increase was directly correlated with the detection of *bla*_*CMY-2*_, a β-lactamase gene. On day 4 post-treatment isolates from two of the treated cows possessed *bla*_*CMY-2*_, while on day 5 four cows did, declining to just one cow by 6 days after treatment. Finally, a recent study in Japan, demonstrated that use of ceftiofur (3^rd^ generation) resulted in excretion of *E*. *coli* resistant to 1^st^ and 2^nd^ generation cephalosporins [33]. Resistant *E*. *coli* contained mutations to the *ampC* gene. Thus, this metagenomic-based study is consistent with what has been observed for several pure cultures, while providing a more comprehensive picture of the antibiotic resistome. Combined, data from the current and published research provide evidence that when cows are treated with ceftiofur, increased fecal shedding of β-lactam-resistant bacteria is a consistent response, at least in the short-term.

### Effect of Ceftiofur on Taxonomic Composition of Cow Feces

**Fig 3:**
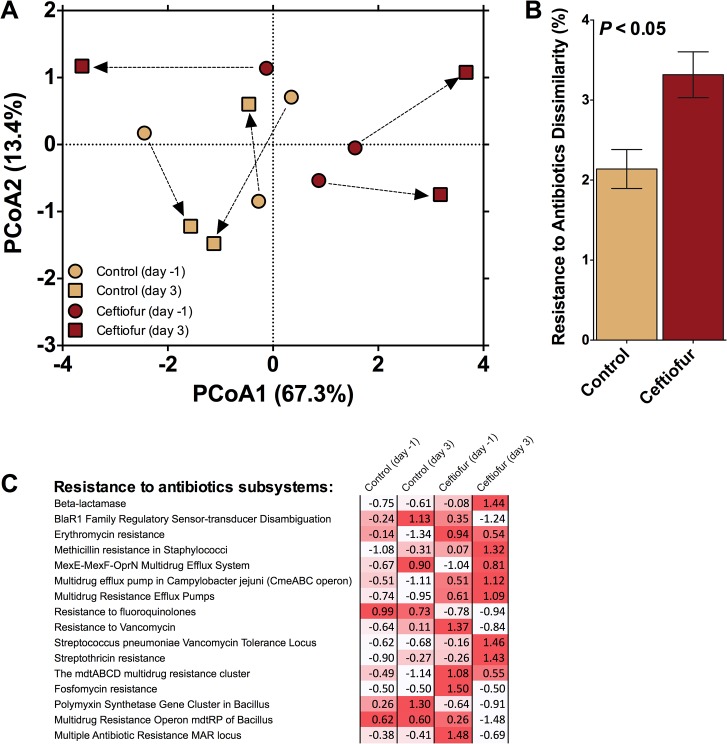
Results examining community composition. A) Principal components analysis illustrating Bray-Curtis distance between initial fecal communities and those after 3 days of either exposure or no exposure to the antibiotic ceftiofur. Arrows indicate the change in communities associated with individual cows. B) Dissimilarity after 3 days between the fecal derived communities of control and ceftiofur treated cows. C) Change in the relative abundance of taxa across treatments. Note that the relative abundance of Class Bacteroidia and Actinobacteria tended to increase and decrease, respectively, after 3 days of exposure ceftiofur.

Taxonomic composition is of interest as an indicator of whether observed shifts in ARG profiles were also associated with shifts in the fecal microbial community structure in response to the antibiotic treatment. PerMANOVA analysis comparing fecal bacterial communities on day 0 and day 3 post-treatment confirmed that significant shifts occurred in the fecal community of both control and ceftiofur-treated cows (*P<*0.10; Fig 3A). No differences in homogeneity of dispersions were noted (*F*_3,8_ = 2.0; *P* = 0.34), indicating that variation across treatments was similar. The difference in community composition appeared to be primarily due to a marked shift in the community associated with ceftiofur-treated cows 3 days post-treatment. In fact, the dissimilarity in communities was greatest for the ceftiofur-treated cows (*P<*0.10; Fig 3B). This indicates that the antibiotic treatment induced a much greater change in fecal bacterial communities when compared to the control (~4% versus ~2.5%, respectively), even after a short three day period. This change, on average, tended to be driven by an increase in the relative abundance of class Bacteroidia and a decrease in class Actinobacteria in ceftiofur-treated cows (Fig 3C). Interestingly, when *Bacteroides* strains (from the class Bacteroidia) were isolated from a variety of human infections, they possessed β-lactamase genes that resulted in reduced susceptibility to a broad spectrum of antibiotics [34]. More specifically, the isolated *Bacteroides* strains were measured to be highly resistant to first- and second-generation cephalosporins and moderately resistant when exposed to third- and fourth-generation cephalosporins[34]. Furthermore, *cfxA* was found to be an essential gene for β-lactamase expression in *Bacteroides* spp. [34], a gene class found to be highly abundant in the feces of ceftiofur-treated cows in the current study. Therefore, there may be a link between the increase in the Bacteroidia class isolated in the manure of ceftiofur-treated cows and the increased resistance associated with β-lactams.

### Functional analysis via MG-RAST

**Fig 4:**
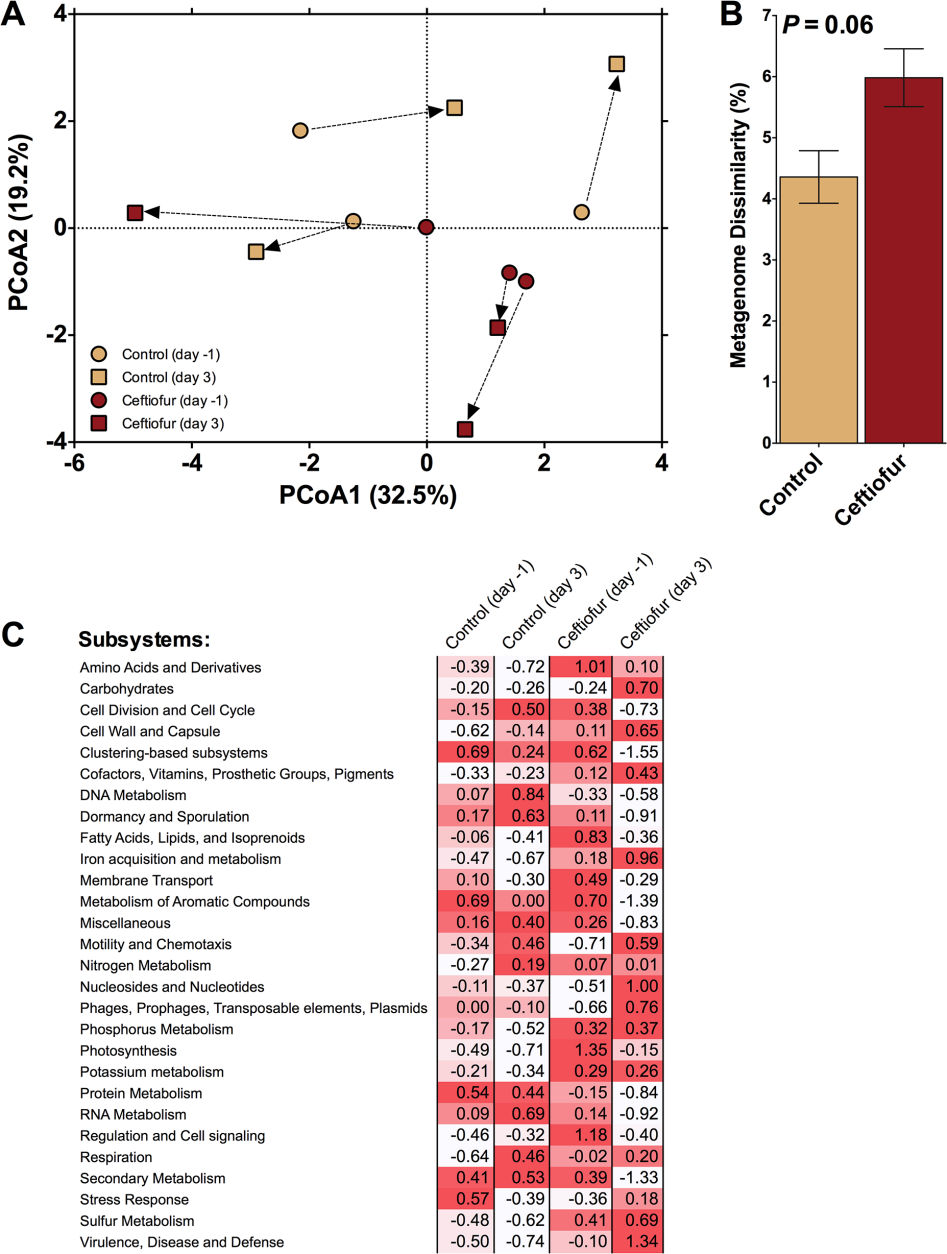
Comparison of metagenomes. A) Principal component analysis illustrating Bray-Curtis distance between initial metagenomes and those after 3 days of either exposure or no exposure to the antibiotic ceftiofur. Arrows indicate the change in communities associated with individual cows. B) Dissimilarity after 3 days between the fecal derived metagenomes of control and ceftiofur treated cows. C) Standard scores (i.e. z-scores) compared across treatments within a given subsystem. Note: Red intensity of z-scores indicates relative increase from the mean associated with that subsystem.

The functional metagenomic composition of the bacterial communities were also altered by ceftiofur treatment, with corresponding shifts in SEED subsystems (P<0.10; Fig 4A). Consistent with the trend observed for taxonomic composition, the dissimilarity in functional metagenomics composition was greatest for the ceftiofur-treated cows (*P<*0.10; Fig 4B), indicating that the antibiotic treatment induced a greater change in the metagenome compared to the control (~6% versus ~4%, respectively). No differences in homogeneity of dispersions were noted (*F*_3,8_ = 2.0; *P* = 0.36), again confirming that variation across treatments was similar. Furthermore, the difference in the ceftiofur-treated cows was driven by change in several functional sub-classifications (Fig 4C), with the most notable change being an average increase in the "Virulence, Disease, and Defense" subsystem.

**Fig 5:**
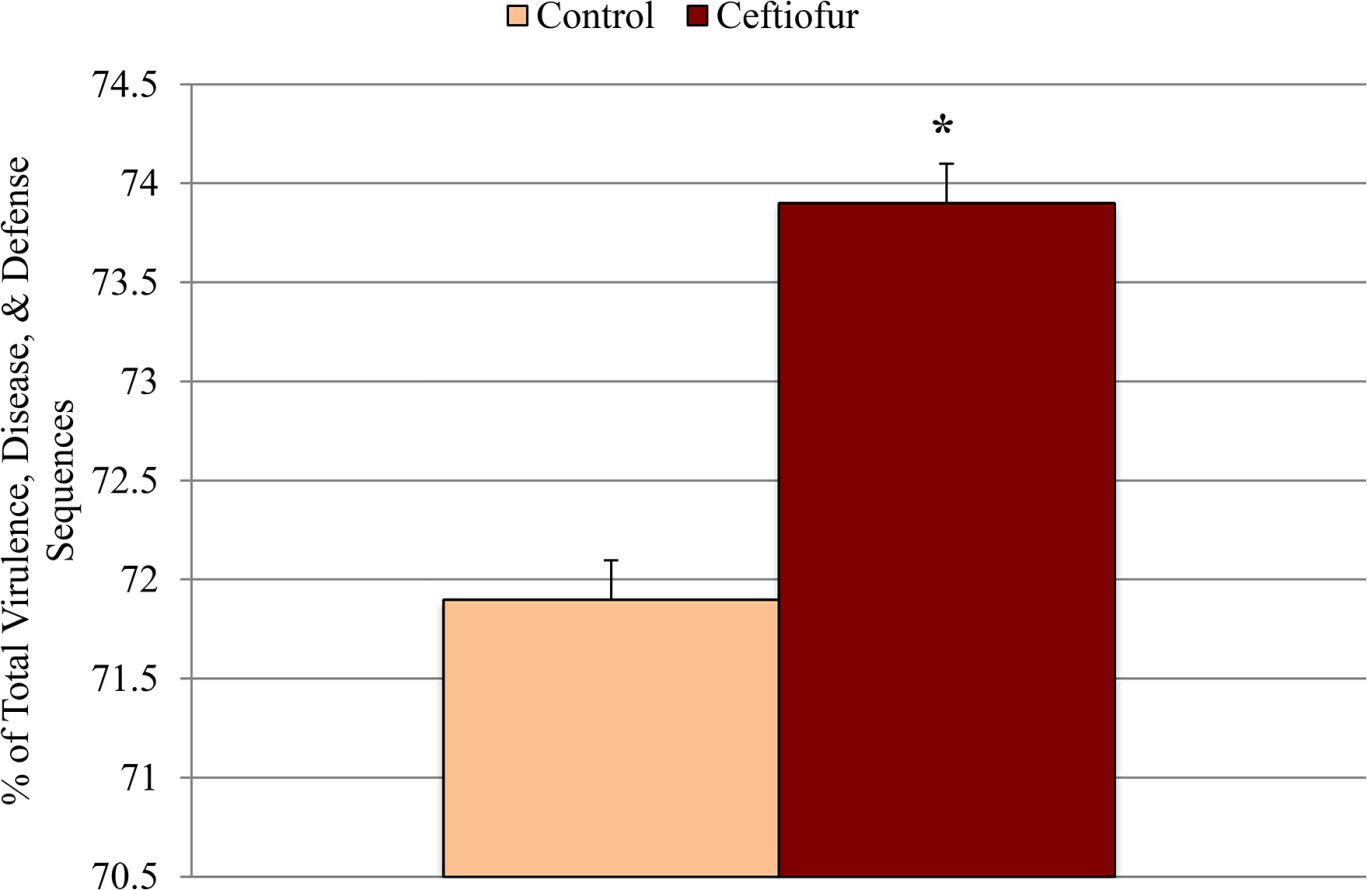
Abundance of “resistance to antibiotics and toxic compounds (RATC)” sequences in fecal samples collected from control (n = 3) and ceftiofur crystalline free acid treated (n = 3) cows on day 3 post-treatment. Day 0 (pre-treatment) samples were used as a covariate. Values are expressed as a percentage of the total “virulence, disease, and defense” sequences. The symbol * indicates significant differences (*P* ≤ 0.10).

**Fig 6:**
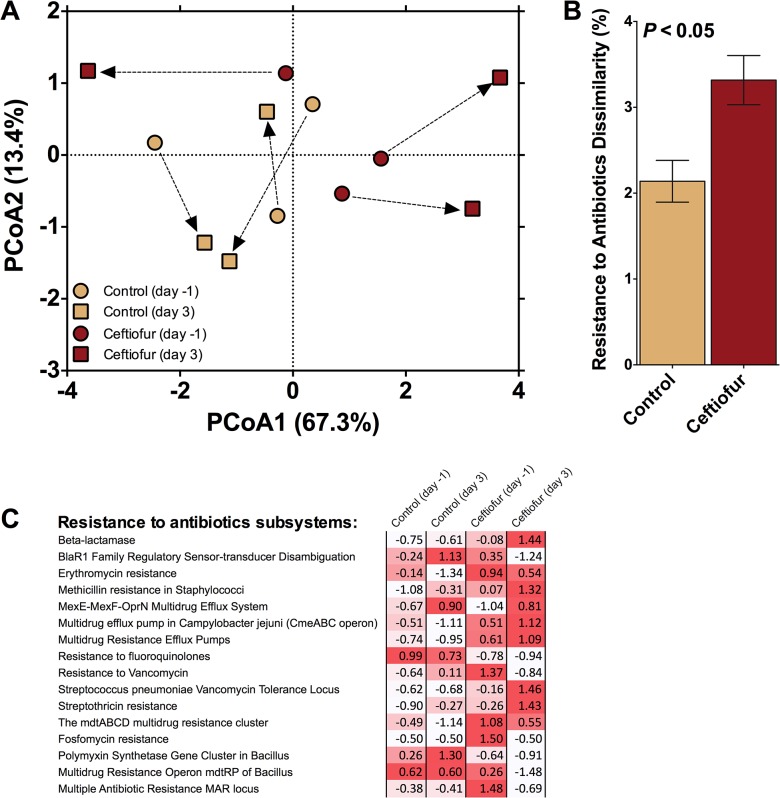
Sequences related to antibiotic resistance found in the 'Resistance to antibiotics and toxic compounds' subsystem. A) Principal component analysis illustrating Bray-Curtis distance between initial sequences related to antibiotic resistance and those after 3 days of either exposure or no exposure to the antibiotic ceftiofur. Arrows indicate the change in communities associated with individual cows. B) Dissimilarity after 3 days between the fecal derived subsystems of control and ceftiofur-treated cows. C) Standard scores (i.e. z-scores) compared across treatments within a given subsystem. Red intensity of Z-scores indicates relative increase from the mean associated with that subsystem.

The abundance of sequences associated with “virulence, disease, and defense” did not differ between control and ceftiofur groups when using day 0 samples as covariate (Table 1). Within this category, however, feces of ceftiofur-treated cows carried a greater proportion of sequences associated with resistance to antibiotics and toxic compounds than control cows (*P* < 0.10; Fig 5). Further examination, focusing only on the composition of the "Resistance to antibiotics" subsystems found under RATC, revealed no significant change in the centroids between treatments (*F*_2,4_ = 0.40; *P* = 0.89) but a significant difference in the homogeneity of dispersions between treatments was noted (*F*_3,8_ = 8.1; *P <* 0.05; Fig 6A). This difference was driven by an increase in dispersion associated with the ceftiofur-treated cows three days after treatment and likely drove the significant dissimilarity observed between control and ceftiofur treatments (Fig 6B). Increased dispersion (*i*.*e*. variation) associated with antibiotic addition may be a response indicative of specific interactions between the host and its microbiome [35]. It is also worth noting that, on average, the day 3 manure from ceftiofur-treated cows tended to exhibit a greater increase in 7 of the 16 "Resistance to antibiotics" subsystems (Fig 6C), than any of the other three treatments.

**Table 1:** Effect of ceftiofur crystalline-free acid antibiotic treatment on the abundance of microbial cell functions in dairy cow feces^a^.

	Treatment			
Functional Category	Antibiotic^b^	Control	SEM^c^	*P* ≤
Amino Acids & Derivatives	9.72	9.65	0.11	0.72
Carbohydrates	16.87	16.78	0.20	0.75
Cell Division & Cell Cycle	1.56	1.60	0.008	0.06
Cell Wall & Capsule	3.74	3.71	0.11	0.87
Clustering-based Subsystems	14.50	14.80	0.03	0.01
Cofactors, Vitamins, Prosthetic Groups, Pigments	4.45	4.48	0.09	0.89
DNA Metabolism	5.16	5.25	0.03	0.12
Dormancy & Sporulation	0.43	0.46	0.01	0.19
Fatty Acids, Lipids, & Isoprenoids	2.01	2.03	0.07	0.84
Iron Acquisition & Metabolism	0.60	0.56	0.03	0.54
Membrane Transport	1.78	1.78	0.03	0.97
Metabolism of Aromatic Compounds	0.44	0.47	0.006	0.06
Miscellaneous	6.25	6.40	0.06	0.19
Motility & Chemotaxis	0.25	0.24	0.003	0.13
Nitrogen Metabolism	0.49	0.50	0.007	0.61
Nucleosides & Nucleotides	3.72	3.50	0.07	0.12
Phages, Prophages, Transposable Elements, Plasmids	3.07	2.34	0.24	0.15
Phosphorus Metabolism	0.51	0.50	0.02	0.74
Photosynthesis	0.02	0.02	0.0006	0.92
Potassium Metabolism	0.11	0.11	0.007	0.72
Protein Metabolism	11.16	11.32	0.13	0.48
RNA Metabolism	6.29	6.45	0.07	0.19
Regulation & Cell Signaling	0.97	0.96	0.01	0.68
Respiration	1.83	1.95	0.06	0.27
Secondary Metabolism	0.20	0.23	0.007	0.07
Stress Response	1.60	1.53	0.02	0.08
Sulfur Metabolism	0.55	1.74	0.03	0.72
Virulence, Disease, & Defense	1.82	1.74	0.03	0.22

Functional sequences expressed as a % of total sample sequences assigned function

^a^n = 3 subcutaneous antibiotic injection, 1.5 mL ceftiofur crystalline free acid sterile suspension (150 mg ceftiofur activity) per 45.4 kg body weight

^b^n = 3

^c^Standard error for LSM

The proportion of sequences associated with “phages, prophages, transposable elements, and plasmids” tended to be higher in ceftiofur-treated cows than in control cows (*P* < 0.15; Table 1). Such sequences are of particular interest given that horizontal gene transfer is a major mechanism of concern for the spread of ARGs [36]. Enrichment of sequences associated with horizontal gene transfer in antibiotic-treated cows suggests selection of the ability to acquire and spread ARGs by ceftiofur [37]. Similarly, when pigs were fed antibiotics (chlortetracycline, sulfamethazine, penicillin), an increase in prophage abundance was detected in the fecal bacteria [38]. Phage activity is known to be a general indicator of transduction, a mechanism of horizontal ARG transfer. Therefore, the observed trend of an increase in these types of sequences may indicate not only the potential to elevate β-lactam ARGs, but also the means to horizontally transfer them to other bacteria.

**Fig 7:**
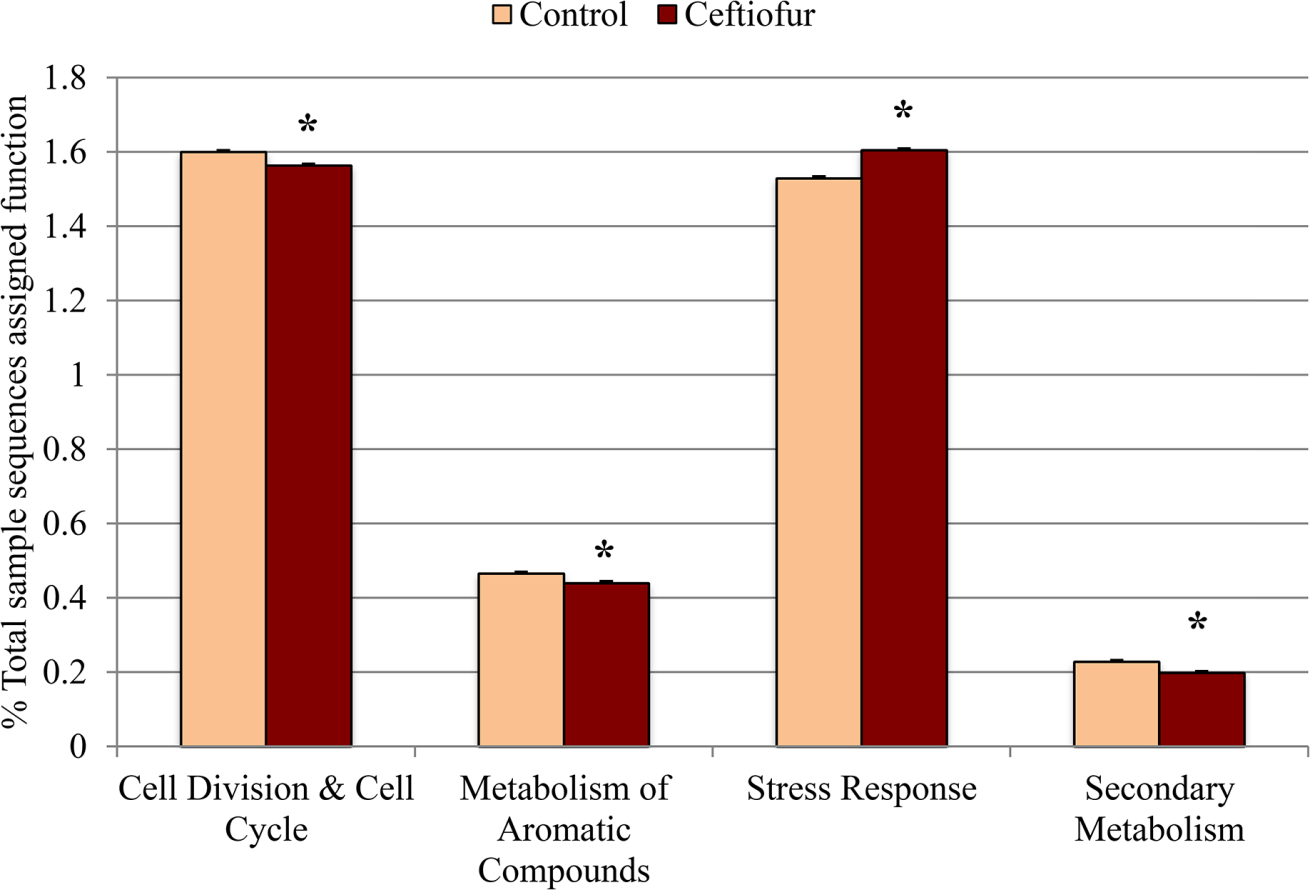
Abundance of “cell division and cell cycle”, “metabolism of aromatic compounds”, “stress response”, and “secondary metabolism” sequences in fecal samples collected from control (n = 3) and ceftiofur crystalline free acid treated (n = 3) cows on day 3 post-treatment. Day 0 (pre-treatment) samples were used as a covariate. Values are expressed as a percentage of the total sample sequences assigned function. The symbol * indicates significant differences (*P* ≤ 0.10).

Ceftiofur-treated cows contained lower (*P* < 0.10) proportion of sequences associated with “cell division and cell cycle” relative to control cows (Fig 7). Reduction in cell division and cell cycle sequences in the bacteria carried by ceftiofur-treated cows can be expected because cephalosporins such as ceftiofur inhibit peptidoglycan synthesis [39], a process critical for cell division. Alternatively, the reduction in cell cycle sequences may actually be advantageous for bacteria capable of surviving under antibiotic stress. The tricarboxylic acid (TCA) cycle plays an important role in producing energy for aerobic and facultative anaerobic bacteria, but TCA mutant bacteria are better able to survive when exposed to β-lactam antibiotics, such as oxacillin [40]. However, the reduced incidence of cell division and cell cycle sequences in the bacteria carried by ceftiofur-treated cows is most likely a negative response to antibiotic pressure rather than a positive one.

The proportion of sequences associated with “metabolism of aromatic compounds” was also lower in ceftiofur-treated cows relative to control cows (*P* < 0.10; Fig 7). Aromatic compounds make up roughly 20% of the earth’s biomass and are mainly produced by plants [41,42]. In particular, lignin is an aromatic compound and a common component of a cow’s plant-based diet (typically 3–4% of the diet). Though aromatic compounds tend to be highly stable, a wide array of bacteria have adapted to their biodegradation and carbon cycling [41]. Therefore, fewer sequences associated with the “metabolism of aromatic compounds” in ceftiofur-treated cows suggests a reduced potential for normal bacterial cell functions, like lignin metabolism, when exposed to antibiotic pressure.

The proportion of sequences associated with “stress response” was higher in the fecal microbiome of ceftiofur-treated cows relative to control cows (*P* < 0.10; Fig 7), consistent with selection of bacteria equipped to cope with stress in the gut of antibiotic-treated cows.

Ceftiofur-treated cows tended to carry a greater (*P* < 0.15) proportion of sequences in their feces associated with bacterial “motility and chemotaxis” than control cows (Table 1). While the exact reason cannot be determined, one possibility is that the increased abundance of sequences associated with motility and chemotaxis in ceftiofur-treated cows incurred a potential increased ability for formation of biofilms, which offer protection against antibiotic exposure. Motility is a key step in the initial stages of biofilm development [43] and chemotaxis factors are also important in biofilm formation, as it is a method bacteria use to attract other bacteria [44].

Ceftiofur-treated cows tended to carry a lower proportion of sequences associated with “secondary metabolism” than control cows (*P* < 0.10; Fig 7). Secondary metabolism involves the natural production of secondary metabolites that often contain antibacterial properties, typically as a response to low nutrient environments [45]. However, this SEED category is not only limited to antibiotic production, but detects other secondary metabolites (e.g. plant hormones like auxin) as well. In general, a lower proportion of sequences associated with “secondary metabolism” in bacteria from ceftiofur-treated cows was likely due to less competition for nutrients as bacteria susceptible to the antibiotic died off.

## Conclusions

Ceftiofur treatment resulted in increases in bacterial sequences associated with resistance to β-lactams and multidrug resistance, indicating that fecal excretion of ARGs by dairy cows is influenced in as little as three days. Further, functional genes associated with horizontal gene transfer were also enriched by ceftiofur, suggesting greater mobility of ARGs selected by antibiotic treatment. Several other functional shifts (e.g., increase in genes associated with stress, chemotaxis, and resistance to toxic compounds; decrease in genes associated with metabolism of aromatic compounds and cell division and cell cycle) were also noted to be associated with ceftiofur treatment, along with measureable taxonomic shifts (increase in Bacterioidia and decrease in Actinobacteria). This study demonstrates that ceftiofur has a broad, measureable and immediate effect on the cow fecal metagenome. Further research is recommended to determine the long-term effect of antibiotic treatment on subsequent fecal shedding of bacterial sequences associated with antibiotic resistance. The metagenomics approach provided a holistic and integrated understanding of ARG responses to antibiotic treatments that are overlooked with single-target methods along with broader implications for animal health and productivity. This study indicates that the costs-benefits of continued use of 3^rd^ generation cephalosporins in dairy cattle should be considered, along with the need for appropriate management of manure to contain the spread of antibiotic resistance.
